# Whole exome and transcriptome sequencing reveal clonal evolution and exhibit immune-related features in metastatic colorectal tumors

**DOI:** 10.1038/s41420-021-00607-9

**Published:** 2021-08-27

**Authors:** Chunxue Li, Juan Xu, Xiangfeng Wang, Chao Zhang, Zicheng Yu, Jiucheng Liu, Zaixian Tai, Ziwen Luo, Xin Yi, Zhaoyang Zhong

**Affiliations:** 1 Department of general surgery, Daping Hospital, Army Medical University (Third Military Medical University), No.10 Changjiang Zhilu, Daping Yuzhong District, 400038 Chongqing, P.R. China; 2 Geneplus Shenzhen, 14 Zhongxing Road, Kengzi Street, Pingshan District, Shenzhen, P.R. China; 3 Taizhou Hospital of Zhejiang Province affiliated to Wenzhou Medical University, No.150 Ximen District, 317000 Linhai, P.R. China; 4 Cancer Center, Daping Hospital, Army Medical University (Third Military Medical University), No.10 Changjiang Zhilu, Daping Yuzhong District, 400038 Chongqing, P.R. China

**Keywords:** Cancer metabolism, Cancer genomics

## Abstract

Liver is the most common site where metastatic lesions of colorectal cancer (CRC) arise. Although researches have shown mutations in driver genes, copy number variations (CNV) and alterations in relevant signaling pathways promoted the tumor evolution and immune escape during colorectal liver metastasis (CLM), the underlying mechanism remains largely elusive. Tumor and matched metastatic tissues were collected from 16 patients diagnosed with colorectal cancer and subjected to whole-exome sequencing (WES) and RNA sequencing (RNA-seq) for studying colorectal cancer clonal evolution and immune escape during CLM. Shared somatic mutations between primary and metastatic tissues with a commonly observed subclonal-clonal (S-C) changing pattern indicated a common clonal origin between two lesions. The recurrent mutations with S-C changing pattern included those in *KRAS, SYNE1, CACNA1H, PCLO, FBXL2, and DNAH11*. The main CNV events underwent clonal-clonal evolution (20q amplification (amp), 17p deletion (del), 18q del and 8p del), subclonal-clonal evolution (8q amp, 13q amp, 8p del) and metastasis-specific evolution (8q amp) during the process of CLM. In addition, we revealed a potential mechanism of tumor cell immune escape by analyzing human leukocytes antigens (HLA) related clonal neoantigens and immune cell components in CLM. Our study proposed a novel liver metastasis-related evolutionary process in colorectal cancer and emphasized the theory of neo-immune escape in colorectal liver metastasis.

## Introduction

The colorectal liver metastasis (CLM) is a multistep and complex process during which tumor cells develop aggressive phenotypic features, and intensely interact with the host immune microenvironment [1]. Approximately 33–50% patients with colorectal cancer (CRC) progressed to CLM [2, 3], which is the major cause of CRC-related deaths. Although previous work tried to reveal the mechanism of CLM at genomic and molecular level, but the large contents were still not completely understood.

Recent researches have highlighted the importance of cancer-specific genetic alterations, mutations in pathways, microsatellite status in predicting CLM, and postoperative survival [4–6]. Yoshikuni et al. analyzed the sequencing data of 1460 patients who underwent CLM resection, and found that multiple somatic mutations in *RAS*, *TP53*, and *SMAD4* were related with worse prognosis [4]. Hu et al. identified the surrogates of metastasis in a cohort enrolling 2751 samples of CRC and suggested that in ~81% patients, tumor cells were commonly disseminated for metastases as early as when the tumor was clinically undetectable (typically, <0.01 cm^3^) [7]. Another study showed that mutations in *APC, KRAS, NRAS, TP53*, or *BRAF* were shared in primary and synchronous liver metastases while *SMAD4* and *PIK3CA* were private driver mutations in metastases [5]. Besides, studies confirmed WNT pathway alterations in CRC including *APC* splice-site alterations in intronic regions and large in-frame deletions in *CTNNB1* [6], alterations in PI3K-Akt signaling pathway and pathways involved in cell adhesion, extracellular matrix (ECM), hepatic stellate activation were found specifically enriched in metastases [8]. However, the clonal evolution process from CRC primary tumor to liver metastases remains largely unclear.

Traditionally, cancer was proposed to be a disease associated with dynamic gene alterations, while increasing studies demonstrated that the immune system also plays a critical role in tumor progression [9]. Immune cells affect the tumor progression and determine tumor cell fate under the strong selection stress [10, 11]. The microenvironment of tumor lesions is a complex system comprising immune cells such as B cell, CD4+ T cell, CD8+ T cell, macrophage and components of fibroblasts, endothelial cells across stages [12]. Among these, tumor-specific neoantigen presentation [13] and T cell response are crucial to eliminate tumor cell, during which human leukocytes antigens (HLA) could present tumor-derived peptides on the cell surface for alpha-beta T cell receptor recognition. Therefore, loss of heterozygosity (LOH) of HLA or unexpression of neoantigens may hinder antigen presentation and facilitate tumor cell immune evasion [14]. The infiltration of CD8+ cells and HLA-I expression at invasive margin were associated with a better overall survival (OS) in metastatic CRC [15].

To obtain comprehensive understanding of the clonal evolution and tumor cell immune escape in course of CLM progress, we performed WES (30 samples) and RNA-seq (27 samples) from 16 patients with CRC and matched liver metastases, and identified new metastasis-associated evolutionary patterns and illuminated tumor immune-related genetic changes in CLM.

## Results

### Somatic mutations and indels in CLM

**Fig. 1:**
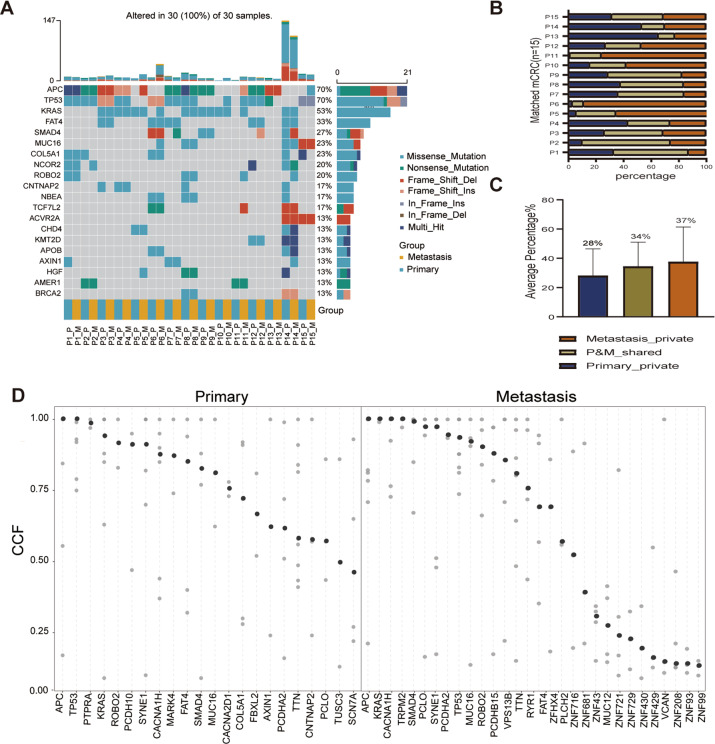
The mutational landscape and proportion and timing order of mutational genes during CLM evolution. **A** Mutations with high mutational frequencies in CRC primary tumors and metastases. **B** The proportion of shared, primary-private and metastasis-private mutations in CRC. **C** The distributions of CCF value of recurrent somatic events in CRC primary and metastases. CCF assessment was performed as described in “Materials and methods.” CLM colorectal liver metastasis, CRC colorectal cancer, CCF cancer cell fraction.

We examined the mutational burdens in 15 pairs of colorectal primary tumors and matched liver metastases and observed 24,590 somatic single nucleotide variations (sSNVs) and 9996 small insertions and deletions (Indels) in those samples. In result, genes with high mutational frequency were components of WNT-β-catenin signaling pathway such as *APC, AXIN1, AMER1, TCF7L2*, and *KMT2D*, mutated in 80% patients (12/15) and genes in HGF/MET signaling pathway including *KRAS, COL5A1* and *HGF*, mutated in 60% patients (9/15) (Fig. 1A). Moreover, the mutations in liver metastases were enriched in ECM, collagen and MET related pathways, which were closely associated with down-stream KRAS-MEK/ERK and PI3K-MTOR signaling pathways (Supplementary Fig. S1B, C). We further analyzed the difference in nonsynonymous SNVs between primary tumors and matched liver metastases of each patient and found that an average of 34% (8–63%) mutations were shared, 28% (1–65%) were primary-private and 34% were metastasis-private (12–88%) (Fig. 1B, C), indicating a common ancestral trunk and genetic heterogeneity between colorectal primary and metastatic lesions. There is an increasing tendency of mutational burdens in metastases compared with the primary tumors. These results suggest that some mutations were newly obtained while some others were lost during the process of tumor evolution. The arm level events of copy number variation (CNV) were showed in matched primary and metastatic lesions (Supplementary Fig. S1A). Major CNVs were identified in most of these lesions, such as 8p del, 8q amp, 13q amp, 17p del, 18q del, 20q amp, and others.

To explore the probable timing order of the mutation events arose in CLM, we analyzed the distribution of the cancer cell fractions (CCF) in samples [16], where high/low median CCF value represents an early or late event, respectively. As shown in Fig. 1C, the median CCF value of *APC* was the highest in both primary tumor and metastases, indicating that *APC* mutation was involved in liver metastasis in addition to tumorigenesis [6]. In addition, we found that median CCF value of *TP53* was higher than *KRAS* in primary tumor, agreeing with the previous conclusion that mutations in *TP53* occur earlier than *KRAS* mutations in primary tumors [17], while *KRAS* mutations occur earlier than *TP53* in liver metastasis. Moreover, in liver metastases, the top-10 mutations with the highest median CCFs were associated with calcium channel or cell-adhesion functions such as *CACNA1H, PCDHA2, PCDHB15, RYR1, FAT4*, indicating tumor cell mobility mediated by Ca^2+^ [18] might play pivotal roles at the early stage of liver metastasis.

### Clonal architecture in CLM

**Fig. 2:**
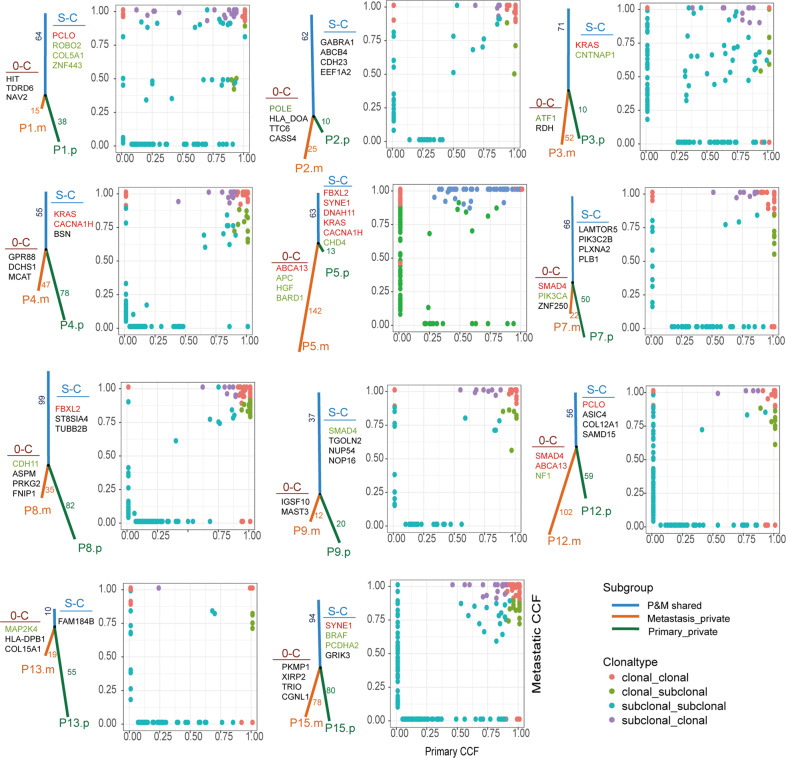
Phylogenetic tree and scatter diagram showing clonal evolution in CLM. The left panel exhibited phylogenetic tree and the right panel exhibited scatter plot in each patient. On the phylogenetic tree, the trunk (blue) indicates mutation shared by both primary tumor and liver metastases, red branch indicates metastasis-private mutations and the green branch indicates primary-private mutations, and the number on trunk or branch indicate amount of corresponding mutations. S-C, subclonal-clonal clonal pattern, 0-C, none-clonal clonal pattern, the genes in green are driver genes and those in red are recurrently mutated genes. m metastasis, p primary. In the scatter diagram, *X*-axis indicates the mutational CCF value in primary tumor, *Y*-axis represents mutational CCF value in liver metastasis, spots with different colors indicate the clonal type from primary tumor to liver metastases: red, clonal_clonal; green, clonal_subclonal; blue, subclonal_subclonal; purple, subclonal_clonal. CLM colorectal liver metastasis, CCF cancer cell fraction.

**Fig. 3:**
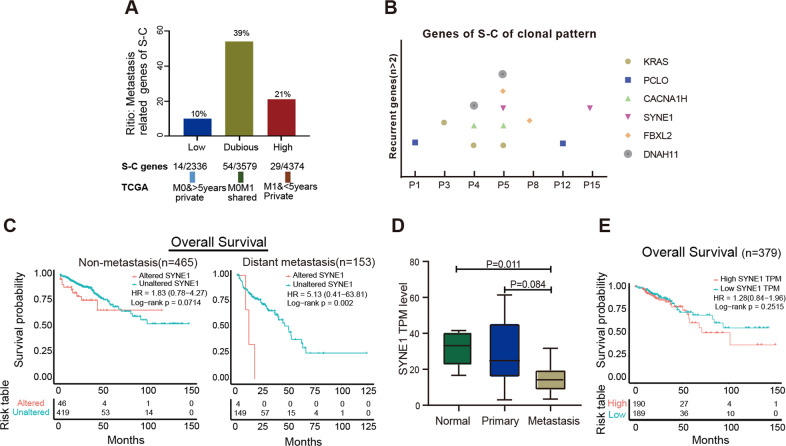
The genes of subclonal_clonal pattern from CRC primary to liver metastasis. **A** The proportion of metastasis-related genes in subclonal_clonal pattern. **B** The recurrent mutations in subclonal_clonal pattern. **C** The correlation between SYNE1 mutations and OS of patients with CRC (**D**), mRNA expression of SYNE1 in normal, CRC primary and liver metastasis. **E** The correlation between SYNE1 mRNA expression and OS of patients with CRC. CRC colorectal cancer, OS overall survival.

We performed phylogenetic analysis based on the nonsynonymous SNVs of primary and metastatic lesions in each patient, and then evaluated precisely the clonality of SNVs by utilizing CCF value [19]. As shown in Fig. 2, the numbers of mutations determined the lengths of corresponding branches and trunks [20]. The overall results agreed with our observations mentioned above, while each patient had individual mutational features. The scatter diagram further displayed the different clonal patterns from primary tumor to liver metastasis in patients. The main clonal evolution patterns, together with corresponding drivers and recurrent mutations (≥2 samples) in S-C models were marked on trunks. The genes with a clonal pattern of subclonal-clonal (S-C) from primary to metastasis were significantly enriched in metastasis-related ECM and collagen-containing ECM pathways (*P* = 0.03) (Supplementary Fig. S2A). To explore if these genes play roles in metastasis, we compared the SNVs in primary lesions between patients without CRC metastasis and with OS of more than 5 years (M0& >5 Y) (*n* = 77) and patients (*n* = 19) with CRC distant metastasis and OS of <5 years (M1& <5 Y) from (TCGA, Firehose Legacy). As shown in Fig. 3A, the private mutations in M1& <5 Y patients were considered as highly metastasis-related, the private genes in patients M0& >5 Y were considered as poorly metastasis-related and shared genes were considered as dubious, respectively. 21% mutations in S-C clonal model were M1& <5 Y-private and 10% mutations of S-C clonal model were M0& >5 Y-private, indicating mutations in S-C clonal model were associated with colorectal distant metastasis. The recurrent mutations of S-C clonal pattern included *KRAS, SYNE1, CACNA1H, PCLO, FBXL2, DNAH11* (Fig. 3B). Among these genes, the mutational frequency of *SYNE1* was 37% (11/30), in only metastatic samples of three patients (P5, P11, P15). Analysis of TCGA (Firehose Legacy) samples showed *SYNE1* mutation was closely associated with metastatic patients’s OS (*n* = 153) (*P* = 0.002) but was not significantly correlated with survival of non-metastatic patients (*n* = 465) (*P* = 0.0714) (Fig. 3C). The mRNA expression level of *SYNE1* in normal, primary and liver metastases gradually decreased, and significantly decreased in liver metastases (*P* = 0.011) (Fig. 3D). However, the level of *SYNE1* mRNA in primary lesions was not correlated with prognosis of patients with CRC by looking at the colorectal cohort (*n* = 379, Firehose Legacy, TCGA) (Fig. 3E).

### Clonal patterns of copy number aberrations in CLM

**Fig. 4:**
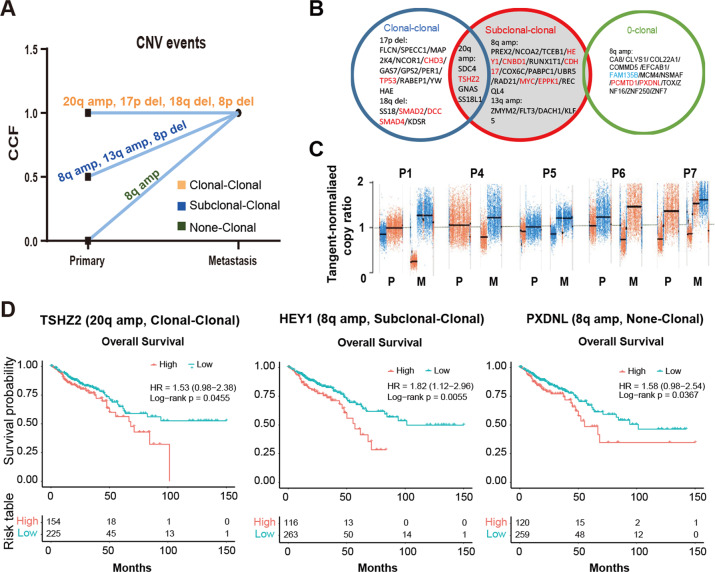
CNV clonal evolution patterns in CLM. **A** The main clonal evolution patterns and corresponding CNV events from CRC primary to liver metastasis. **B** The driver genes on segments with various CNV clonal evolutionary patterns. **C** The somatic copy number alterations of Chromosome 8q in matched primary tumor tissues and metastatic tissue in five patients; copy ratios were calculated by GATK software. **D** The genes associated with CRC prognosis on segments with CNV events. CNV copy number variation, CLM colorectal liver metastasis, CRC colorectal cancer.

We further compared the copy number variations (CNVs) between primary tumors and liver metastases. The evolutionary patterns from primary to liver metastases were shown in Fig. 4A, where chromosomes(Chr) 20q amplification(amp), 17p deletion(del), 18q del, 8p del were clonal-clonal(C-C), 8q amp, 13q amp, 8p del were S-C and 8q amp was non-clonal (0-C) according to the CCF of these segmental aberrations, indicating that Chr 20q amp, 17p del, 18q del, 8p del were considered as the early events, 8q amp was the liver metastasis-private later events, and 8q amp, 13q amp, 8p del were considered as middle-stage events. The driver genes in these segments including *TP53* (17p del), *SMAD2, SMAD4*(18q del) and *MYC, CNBD1, HEY1, RUNX1T1, CDH17*(8q amp) (Fig. 4B). High mRNA expression level of *TSHZ2* or *HEY1* was associated with poor outcome in colorectal cohort (TCGA, Firehose Legacy) (Fig. 4D). In addition, clonal metastasis-private Chr 8q amplification was observed in patient 1, 4, 5, 6 and 7 among all (Fig. 4C). The frequencies of focal CNVs on this arm were significantly different between primary tumor and metastasis, spanning *PXDNL*, the high expression level of *PXDNL* was related to poor OS in colorectal cohort (TCGA, Firehose Legacy) (Fig. 4D). These results indicated the clonal evolution of CNV across CLM stages, including the amplification of Chr 8q and corresponding genes were crucial events for late liver metastasis.

### HLA-LOH related TMB and neoantigens in CLM

**Fig. 5:**
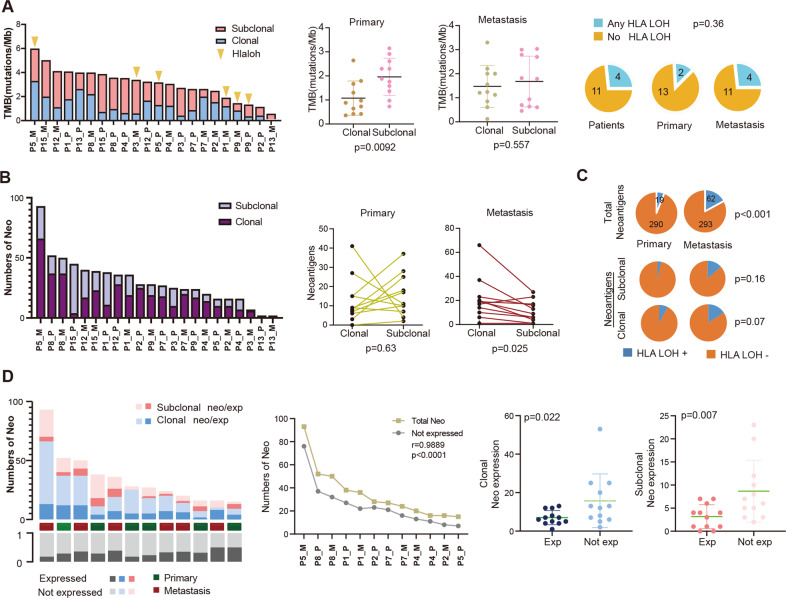
HLALOH-related TMB and neoantigens in CLM. **A** The distribution of mutation clonality and HLA LOH events. **B** The distribution of neoantigens clonality. **C** The distribution of HLALOH events in total neoantigens, clonal neoantigens and subcloanal neoantigens. **D** Neoantigen depletion in transcriptional level. Top, the clonal and subclonal expressed (exp) neoantigens (neo) in each patient. Bottom, the fraction of clonal neoantigens that are ubiquitously expressed. Correlation analysis between the counts of neoantigens and non-expressed neoantigens (middle panel). The counts of clonal or subclonal neoantigen depletion (right two panels). TMB Tumor mutation burden, HLA Human leukocytes antigens, LOH Loss of heterozygosity.

Tumor mutation burden (TMB) is an indicator to evaluate the efficacy of immunotherapy [21], but a new concept of human leukocyte antigen (HLA)-corrected TMB was proposed, which considered tumor-specific neoantigen presentation hindered due to LOH of HLA in tumor immune response [14, 22]. We then examined the subclonal and clonal TMB and distribution of HLA-LOH (Fig. 5A). In result, subclonal mutation load was considerably higher (*P* = 0.009) than clonal in primary lesions, while in liver metastases, subclonal and clonal mutation loads had no significant difference (*P* = 0.557). One possible reason is the bottleneck effects in the process of tumor evolution and selection during metastasis. The proportion of clones mutation was increased compared to subclonal mutation in metastatic lesions due to metastasis specifically selected certain tumor clones. Subclonal mutation loads in primary tumors were higher than metastasis while the clonal mutation loads were lower (Supplementary Fig. 3A), this trend is agreeing with the results of S-C evolutionary pattern from primary to liver metastasis. The positive incidence of LOHHLA was 26% (4/15) in all patients, 13% (2/15) in primary tumors, 26% (4/15) in liver metastases. The positive incidence of LOHHLA in primary tumors and liver metastases has no significant difference (*P* = 0.36), while LOHHLA in liver metastases has increased tendency. Although both TMB and HLA-corrected TMB had no significant difference between primary and liver metastasis, HLA-corrected TMB increased the *P* value from 0.41 to 0.26 (Supplementary Fig. 3B).

The distribution of clonal/subclonal neoantigens in all samples were shown in Fig. 5B, there was no statistical difference between the number of clonal and that of subclonal neoantigens in primary tumors (*P* = 0.62), while the count of clonal neoantigens was higher than subclonal in liver metastasis (*P* = 0.0025). Moreover, the prevalence of neoantigen-located LOHHLA of liver metastases was significantly higher than in primary tumors (*P* < 0.001). Also, in liver metastases, numbers of clonal and subclonal neoantigens increased compared with primary tumors (Fig. 5C). The mRNA expression of neoantigen is shown in Fig. 5D, where the number of neoantigens was positively correlated with the number of non-expressed neoantigens (*r* = 0.9889, *P* < 0.0001). The numbers of non-expressed neoantigens in both clonal and subclonal groups were significantly higher than that of expressed neoantigens, especially in the subclonal group (*P* = 0.007). Taken together, under the pressure of immune selection and seeding, the HLALOH and the deletion of expression of neoantigens occurred during the process of tumor evolution and metastasis.

### Immune cell micro-environment in CLM

**Fig. 6:**
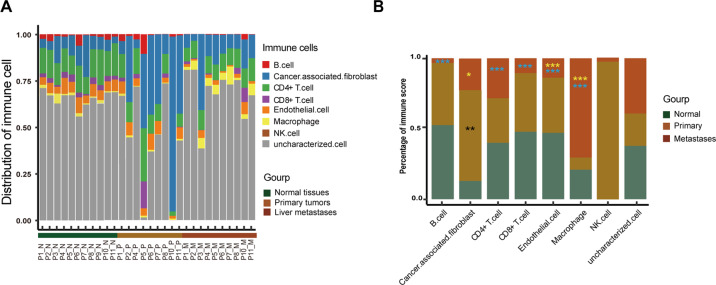
The distribution of immune cell in various stages of CRC. **A** The distribution of B cell, cancer associated fibroblast, CD4+ T cell, CD8+ T cell, endothelial cell, macrophage, NK Cell and uncharacterized immune cell in normal, CRC primary tumor and liver metastasis. **B** The difference of immune infiltration in various stages of CRC. Yellow asterisk (*) indicates the significant difference between metastasis and primary, blue asterisk (*) indicate the significant difference between normal and metastasis, black asterisk (*) indicates the significant difference between normal and primary tumors. *p < 0.05, **p < 0.01, ***p < 0.001.

To explore if the immune-cell micro-environment affects CLM progress, we used RNA-seq to analyze the distribution of immune cells in normal, primary and matched liver metastatic tissues. The different proportions of immune cells including B cells, cancer associated fibroblasts, CD4+ T cells, CD8+ T cells, endothelial cells, macrophages in normal, primary and matched liver metastasis tissues reflect the different immune microenvironment status across stages of tumor progression (Fig. 6A). B cells were mainly enriched in normal tissues, while the number of B cells reduced significantly in liver metastases (*P* *<* 0.001), indicating human immunity defects in liver metastases; primary tumor had the most cancer associated fibroblasts compared to normal and metastatic samples (*P* = 0.001, *P* = 0.015); CD4+ T cells, CD8+ T cells and endothelial cells were significantly decreased in liver metastasis than in normal tissues or primary tumors (*p* < 0.001), while macrophages increased significantly in liver metastasis (*p* < 0.001) (Fig. 6B). These results suggested that immune response was downregulated due to reduced CD4+ /CD8+ cells infiltration in liver metastases, while tumor-associated macrophages (TAMs) remained at a high level.

## Discussion

Tumor malignant progression is a dynamic evolutionary process, researchers sought to explore the genetic features of tumors across various stages [23], distinguishing the early or later events during malignant progression [16], and tried to profile genetic variations [24] and micro-environment immune architectures to explain how tumor cells escape the immune surveillance in metastasis progress [25]. In our study, we performed a multi-level investigation to understand the CLM process by proposing a new metastasis-related clonal evolutionary pattern, and determined the crucial corresponding genes and clonal CNV events in this pattern. We also integrated transcriptional analysis of mRNA to explore the tumor cell immune escape based on HLALOH-related neoantigens and elaborate immune cell microenvironment in course of CLM.

In our study, CRC primary and liver metastasis had the common ancestor at the mutational level, meanwhile had their private features (Fig. 1A). We found the conventional tumorigenesis-related WNT signaling pathway would have potential abilities of inducing CLM (Fig. 1B, Supplementary Fig. 1A), in CRC patient P5, P11, P15, *APC* mutation was only observed in the metastatic samples, and CCF analysis showed *APC* mutation was an early event in liver metastasis, indicating that *APC* would have a function of facilitating liver metastasis. The average percentage of liver metastasis-private mutation is higher than of primary-private mutations, this tendency was consistent to what has been previously described [8], however our data showed that liver metastasis-related signaling pathways include HGF/MET and its down-stream KRAS-MEK/ERK or PIK3CA-MTOR signaling pathway, which affect the proliferation, angiogenesis and migration of tumor cells [26, 27], not just PI3k-AKT or TGF-β signaling pathway. In addition, we firstly proposed that earlier events in metastatic lesions are enriched in cell adhesion and Ca^2+^ channel functions, including mutations in *CACNA1H, PCDHA2, PCDHB15, RYR1,* and *FAT4*, indicating the potential components of cell mobility in early liver metastasis.

One highlight in our study is the finding that mutated genes with S-C clonal evolution from CRC primary tumor to liver metastases are enriched in cell mobility-related ECM signaling pathways and were associated with metastasis by cohort analysis (Figs. 2,3). Subclonal mutations were the later events compared to clonal mutations in primary tumors and played essential role in the manifestation of intratumoral heterogeneity. We speculated these ECM-related subclones exert important functions in changes of microenvironment to promote tumor cell invasion and metastasis. A “big bang” model proposed the theory of ‘born to be bad’ metastatic pattern in CRC, in which researchers emphasized the determined exist of the subclone private alterations in early malignant in the final neoplasms [28]. *SYNE1*, a mutated gene with S-C clonal evolution, had a high prevalence of mutation in fuctional region which was observed only in liver metastases in 20% patients with CRC and lower expression in liver metastatic lesions. However, the *SYNE1* mRNA level in primary CRC was not correlated with unfavorable prognosis, indicating functional mechanism of *SYNE1* in CLM was obscure, whether there was synergistic effect between gene mutation or protein level of *SYNE1* in CLM process should be further investigated in future studies. Other studies have also confirmed that abnormal methylation of *SYNE1* promoter is closely related to CRC [29], and is a promising marker for CRC detection [30].

In CNV events level, we firstly confirmed the different clonal patterns of major CNV events. Moreover, we explored the probable timing order of the CNV events from CRC primary to matched liver metastases according to CNV clonality. Clonal pattern of C-C were early events, 0-C defined as liver metastasis-private were later events, and S-C were defined as middle-stage events (Fig. 4A). Among early CNV events, the deletion of Chr 18q was correlated with hepatic metastasis [31], driver genes *SMAD2* and *SMAD4* were located on Chr 18q. Another early event is Chr 17q del and *TP53* is a key driver gene on this segment, *TP53* is a prevalently mutated gene and closely associated with CRC malignancy [32]. The corresponding genes of middle-stage CNV events including *MYC, CNBD1, HEY1, RUNX1T1, CDH17*, high expression of *HEY1* gene promotes self-renewal of tumor stem cells, especially in liver cancer [33]. On liver metastasis-private later-stage CNV chr8q, the gene *PXDNL* with significantly different mutational frequency was associated with the CRC prognosis. These finding enriched the knowledge for understanding CLM progression.

At present, it is difficult to completely understand the process of immune escape of tumor cells in CLM. Immune microenvironments were varied both between and within tumors as well as in various stages during tumor evolution. We found the incidence of HLALOH and the number of clonal neoantigens significantly increased in liver metastasis. The HLALOH and deletion of expressed neoantigens may be a potential mechanism of CRC cell immune escape in agree with Rosenthal R et al.’s report in lung cancer [13]. However, the evidence of immune escape due to HLA-LOH is not enough in the present study, given that most analyses were association tests, and limited analyses yielded statistical significance. Modest sample size might be the reason leading to low statistical significance, and since our study was based on biopsy samples from clinical practice, we might not be able to perform further mechanistic analyses. Studies with larger sample sizes and various approaches are needed to elucidate the mechanisms behind the immune escape of the metastasis of colorectal tumors. It is notable that HLALOH occurred in the samples with recurrent mutations of S-C clonal pattern including *KRAS, SYNE1, FBXL2, DNAH11 and CACNA1H*, indicating this mutational clonal patter is facilitating tumor cell to escape immune monitor. Besides, the different CRC stages have distinct immune microenvironment, the CD4+, CD8+ T cells were significantly decreased and TAMs significantly increased in liver metastases, indicating T cell mediating immune responses was inhibited when TAM induced tumor cell metastasis by promoting angiogenesis [34], and promoted tumor cells disseminated to extravasation and survival by inhibiting immune-mediated elimination [35]. In addition, hepatic stellate cells can secrete cytokines like HGF, and TGF-β to degrade ECM, which also stimulates angiogenesis and inhibits the immune response [36]. Taken together, by exhibiting the TMB and HLA-LOH features of metastatic lesions and primary tumors in CRC, we could provide with a more comprehensive landscape and systematic report to gain a deeper insight into the genetic underpinnings of CRC metastasis, and offer essential referencing information to guide the use of immune therapies and other treatment decisions.

In summary, our data exhibited a new metastasis-associated clonal evolutionary pattern at somatic SNV and CNV levels, revealed immune microenvironment in CLM and explored the potential mechanism of immune escape via LOH of HLA. Our results provide a new experimental basis for understanding CLM.

## Materials and methods

### Data sources and sample information

Sixteen patients with CRC were enrolled from Daping Hospital, Army Medical University (Third Military Medical University) from 2015 to 2019. Patients were either untreated or treated with neoadjuvant therapy containing oxaliplatin, Irinotecan or bevacizumab. The detailed clinicopathological and sample information were shown in Supplementary Table 1 (one tumor sample without gene mutation was removed from the WES queue). Written informed consent was granted in sample collection, data analysis and publication. This study was approved by the institutional review board of Daping Hospital, Army Medical University.

Online analysis of TCGA datasets including clinical and SNVs data of 77 patients (1). without CRC metastasis and (2). with OS of more than 5 years (M0& >5 Y), 19 patients with (1). CRC distant metastasis and (2). OS of <5 years (M1& <5 Y), and Clinical and SNVs data of *SYNE1* mutated CRC patients were obtained from public databases of The Cancer Genome Atlas (TCGA, Firehose Legacy) (http://www.cbioportal.org; http://gdac.broadinstitute.org) and analyzed.

### Sample treatment, target enrichment, and whole-exome sequencing

Primary tumor with matched normal and liver metastasis tissues were collected and prepared into formalin-fixed paraffin-embedded (FFPE) samples. Genomic DNA extraction was performed with the TIANamp Genomic DNA kit (Tiangen Biotech, Beijing, China) following manufacturer’s instruction. The purity and concentration of DNA were determined using Nanodrop 2000 spectrophotometer and Qubit 2.0 Fluorometer with Quanti-IT dsDNA HS Assay Kit (Thermo Fisher Scientific, MA, USA). Library construction was then performed using a custom 53 M length capturing probe, made by Integrated DNA Technologies (IDT, IA, USA), and covering the coding regions of all genes and partial non-coding regions. Captured libraries were then pair-end sequenced in 100 bp lengths with Geneplus-2000 sequencing platform (Geneplus, Beijing, China) following the manufacturer’s guidance. Raw data from next-generation sequencing was then filtered to remove low-quality reads and adaptor sequence. Reads were further mapped to the reference human genome (hg19) utilizing BWA aligner (version 0.7.10) for mutation calling. In total, 15 pairs of primary and metastasis samples were subjected to WES with a mean sequencing depth of 150×. Matched DNA from normal colorectal tissue was used as control.

### Clonality evaluation of SNVs and SCNAs

The software of ABSOLUTE (version 1.2) was used to evaluate the tumor purity and genomic ploidy by analyzing CCF and allele-specific copy-numbers of each sample [37]. To ensure the accuracy of clonal inference, samples with a low tumor cell purity (below 20%) were excluded from further analysis. SNVs and SCNAs were defined as clonal if Maximum CI-95% of CCF ≥ 0.95 (ccf_CI95_high ≥ 1) and probability of clonal mutations is higher than subclonal mutations (Pr_somatic_clonal > Pr_subclonal), otherwise were defined as subclonal.

### Phylogenetic tree reconstruction

We used PHYLIP (version 3.6) (https://evolution.genetics.washington.edu/phylip/phylipweb.html) with the maximum parsimony method to infer the phylogeny of multiple specimens from individual patients based on the presence or absence of SNVs and indels. FigTree (version 1.4.3) (http://tree.bio.ed.ac.uk/software/Figtree/) was used to visualize the reconstructed trees.

### Neoantigen prediction and expression of neoantigen

To screen the neoantigen, we employed Depth based filters as follows [38], any variants with normal coverage ≤ 5× and normal VAF of ≥2% were filtered out. The normal coverage cutoff can be increased up to 20× to eliminate occasional misclassification of germline variants as somatic. For tumor coverage from DNA or RNA, a cutoff is placed at ≥10× with a VAF of ≥40%. To further evaluate the effect of relevant nearby variants on neoantigen identification, we used netMHC- 4.034 an updated version of the pVAC tools software to assess the binding affinities of the neoantigens with the corrected mutant peptide sequence [39]. When RNA-seq data were available, a neoantigen was considered to be expressed if at least five RNA-seq reads could be mapped to the mutation site, and at least three contained the mutated base [13].

### Identifying HLALOH event in tumors

The HLA LOH events in tumor were identified with the method by McGranahan N. et al., HLA-LOH was defined when one of the two alleles of HLA gene was below 0.5, and there was significant difference between the log copy ratio of the two alleles (*P* Val_unique < 0.01) [14].

### Sample treatment and RNA seqencing

Primary tumor with matched normal and liver metastasis tissues were collected and prepared into FFPE samples. RNA extraction, sequencing library construction, sequencing and FASTQ data quality control were performed in accordance with the protocol by Nick D.L. Owens et al. [40], alterations were then matched to the hg19 genome using STAR software [41].

### Estimating immune cell populations

Based on RNA-seq data, we calculated the immune cell scores in normal, primary and matched liver metastasis region to explore the different immune microenvironment status and immune infiltration in these tissues with the method stated in https://icbi-lab.github.io/immunedeconv/ website [42]. The immune cell populations considered included B cell, cancer associated fibroblast, CD4+ T cell, CD8+ T cell, endothelial cell, macrophage and NK cell.

### Statistics

Chi-Square test or the Fisher’s exact test was employed to calculate the significant difference in categorical variables between two groups, while Wilcoxon Mann–Whitney test was performed for comparing continuous variables. R software (version 3.6.1), Linux sever equipped with Python (version 2.7.13) or GraphPad Prism software (version 8.0.2) were employed to analyze data and carry out visualization. Statistical significance was defined as *P* < 0.05 for paired two-tail student *t* tests.

## Supplementary information

supplementary table 1

Figure S1

Figure S2

Figure S3

Supplementary figure legends

## Data Availability

The sequencing data have been deposited in China National GeneBank DataBase under the project number CNP0001817.
