# Supplementary material for: Whole exome and transcriptome sequencing reveal clonal evolution and exhibit immune-related features in metastatic colorectal tumors
Source: Cell Death Discov. 2021 Aug 27;7:222. doi: 10.1038/s41420-021-00607-9 (PMC8397721; doi:10.1038/s41420-021-00607-9)
Supplement: Supplementary file 1 — supplementary table 1 [file 41420_2021_607_MOESM1_ESM.docx]

| **Suppl Table 1 CRC Patient clinical and sample information** | | | | | | | | | | | | | | | | | | |
| --- | --- | --- | --- | --- | --- | --- | --- | --- | --- | --- | --- | --- | --- | --- | --- | --- | --- | --- |
| **Sample** | **Sex** | **Age** | **Clinical TNM** | **Clinical stage** | **Pre-surgical therapy** | **Therapeutic regimen (time order from left to right)** | **Primary lesions** | **Gross type of tissue** | **Histologic pathology** | **Pathologic TNM** | **Pathologic stage** | **Lymph node +** | **Metastatic sites** | **Liver postoperative pathology** | **Primary purity** | **Metastatic purity** | **Primary ploidy** | **Metastatic ploidy** |
| P1 | F | 68 | T4N2M0 | ⅢC | - | Surgery (p), chemo, surgery (m) | colon | Mass | Middle ACC | T4N2M0 | ⅢC | 10/14 | liver | RL: Moderately differentiated ACC | 0.56 | 0.84 | 1.69 | 1.91 |
| P2 | F | 47 | T4N1M1 | Ⅳ | - | Surgery (p&m), chemo, surgery (m) | rectum | ulcerative | Middle ACC | T4N0M1 | Ⅳ | 0/13 | liver | Ll: Moderately differentiated ACC | 0.34 | 0.55 | 3.01 | 3.13 |
| P3 | F | 48 | T4N1M1 | Ⅳ | - | Surgery (p&m), chemo combined perfusion chemo | rectum | ulcerative | Middle ACC | T4N1M1 | Ⅳ | 2/22 | liver | RL: Moderately differentiated ACC | 0.32 | 0.14 | 2.61 | 2.19 |
| P4 | F | 47 | T3N2M0 | ⅢB | Neoadjuvant | Neoadjuvant, surgery (p), CRT, surgery (m), chemo | rectum | Mass | Middle ACC | T3N2M0 | ⅢB | 3/12 | liver | RL: ACC | 0.66 | 0.49 | 1.88 | 1.93 |
| P5 | M | 59 | T3N1M0 | ⅢB | Neoadjuvant | Neoadjuvant, radiotherapy, surgery (p), chemo | rectum | ulcerative | Middle ACC | T2N0M0 | Ⅰ | 0/4 | liver | RL: ACC | 0.23 | 0.44 | 2.89 | 2.1 |
| P6 | F | 57 | T4N2M1 | Ⅳ | - | Surgery(p), Surgery(m), chemot | rectum | ulcerative | Middle ACC | T4N0M1 | Ⅳ | 0/13 | liver | LL: Moderately differentiated ACC | 0.39 | 0.59 | 2.02 | 1.98 |
| P7 | M | 70 | T4N0M0 | ⅡB | - | Surgery(p), chemo, surgery(m), chemo | colon | ulcerative | Middle ACC | T4N0M0 | ⅡB | 0/19 | liver | LL: Moderately differentiated ACC with necrosis | 0.38 | 0.36 | 2.64 | 2.49 |
| P8 | M | 69 | T4N2M0 | ⅢC | - | Surgery(p), chemo, surgery(m) | colon | ulcerative | Middle ACC | T4N2M0 | ⅢC | 7/10 | liver | RL: Moderately differentiated ACC | 0.63 | 0.73 | 1.94 | 1.92 |
| P9 | M | 48 | T3N2M1 | Ⅳ | Neoadjuvant | Perfusion chemo& RAT, surgery(p), surgery(m) | rectum | ulcerative | Middle ACC | T2N0M1 | Ⅳ | 0/12 | liver | RL: Moderately differentiated ACC | 0.29 | 0.32 | 2.03 | 2 |
| P10 | M | 44 | T3N2MX | ⅢB | Neoadjuvant | Chemo, perfusion chemo, surgery(p), chemo | rectum | ulcerative | Middle ACC | T3N1M1 | Ⅳ | 1/12 | liver | RL: Moderately differentiated ACC | 0.95 | 0.23 | 2.07 | 2 |
| P11 | M | 53 | T4bNxM1 | Ⅳ | Neoadjuvant | Chemo, CRT, surgery(p),chemo, surgery(m), chemo | rectum | ulcerative | Middle ACC | T2N2bM1 | Ⅳ | 7/15 | liver | Moderately differentiated ACC with necrosis | 0.96 | 0.47 | 2.12 | 2 |
| P12 | M | 42 | T4N2M1 | Ⅳ | - | Surgery(p), chemo, surgery(m), chemo, surgery(m) | rectum | ulcerative | Middle ACC | T4N2M1 | Ⅳ | 5/9 | liver | - | 0.77 | 0.68 | 2.67 | 2.97 |
| P13 | M | 59 | T4N0M1 | Ⅳ | - | Surgery(p), chemo | rectum | - | Middle ACC | T4N0M1 | Ⅳ | - | liver | - | 0.89 | 0.59 | 1.88 | 1.77 |
| P14 | M | 41 | T4N1M1 | IV | - | Surgery(p), chemo | colon | ulcerative | Middle ACC | T4N1M1 | IV | 3/8 | liver | - | 0.67 | 0.58 | 2 | 2.04 |
| P15 | M | 65 | T3N0M1 | Ⅳ | - | Surgery(p), chemo, CRT,Chemo,Surgery(m) | colon | - | Middle ACC | T3N0M1 | IV | - | liver | - | 0.89 | 0.46 | 1.84 | 1.85 |

F, female; M, male; p, primary; m, metastasis; CRT, Chemoradiotherapy; P&M, primary and metastasis; ACC, adenocarcinoma; RL, right liver; LL, left liver.
