# Supplementary figures and images for: Whole exome and transcriptome sequencing reveal clonal evolution and exhibit immune-related features in metastatic colorectal tumors
Source: Cell Death Discov. 2021 Aug 27;7:222. doi: 10.1038/s41420-021-00607-9 (PMC8397721; doi:10.1038/s41420-021-00607-9)

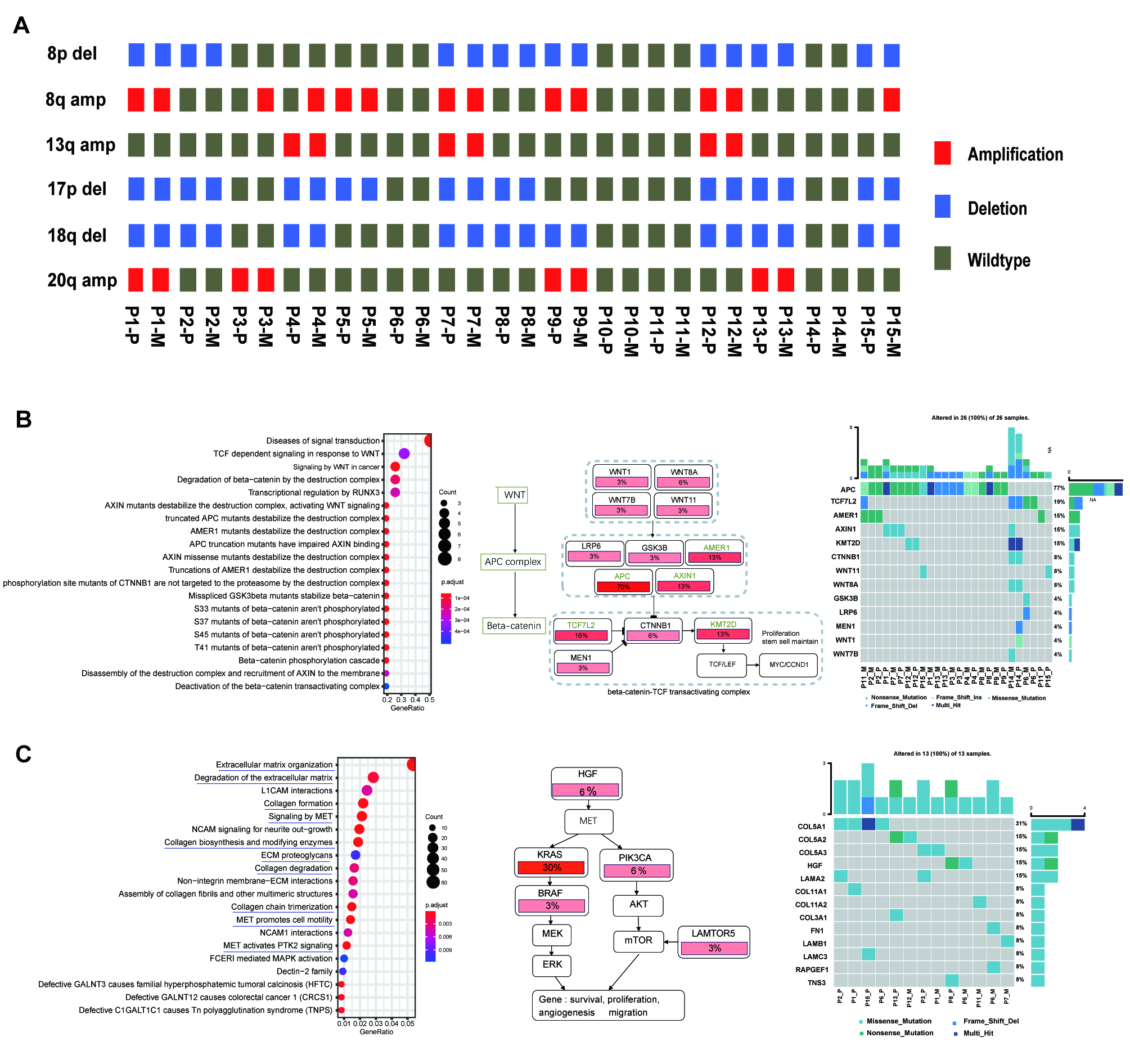

Supplement: Supplementary file 2 — Figure S1 [file 41420_2021_607_MOESM2_ESM.tif]

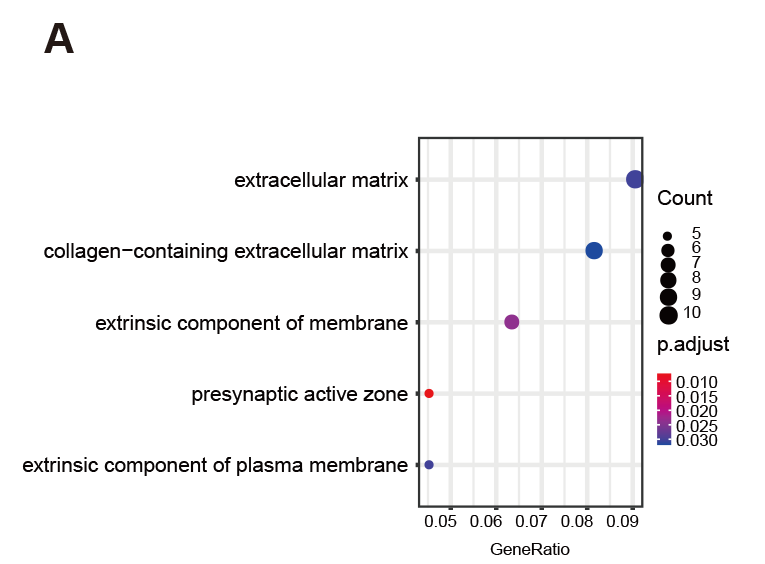

Supplement: Supplementary file 3 — Figure S2 [file 41420_2021_607_MOESM3_ESM.tif]

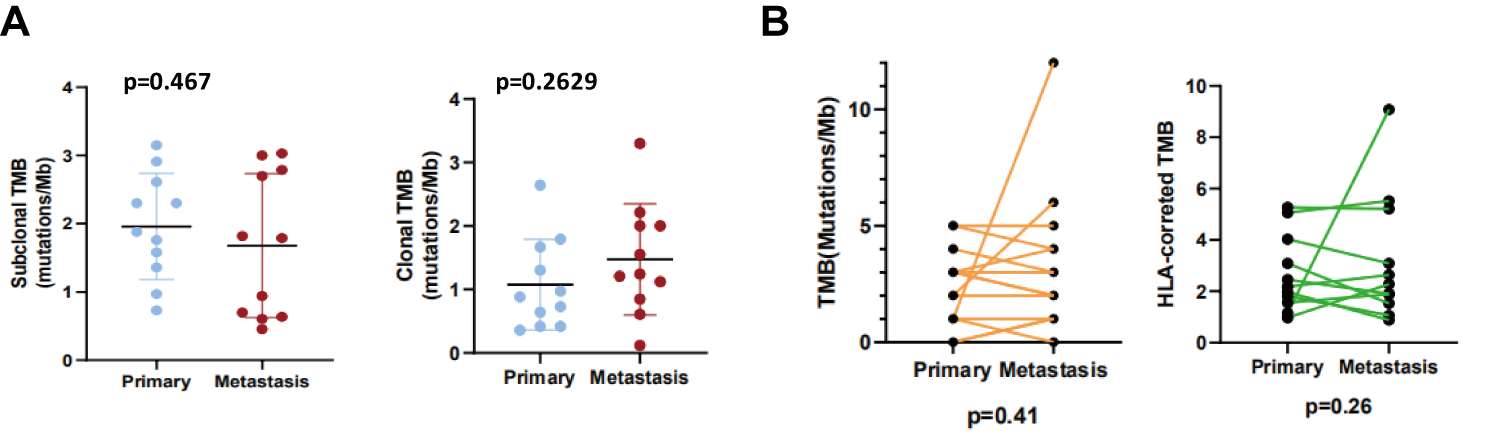

Supplement: Supplementary file 4 — Figure S3 [file 41420_2021_607_MOESM4_ESM.tif]
