# Supplementary material for: Whole exome and transcriptome sequencing reveal clonal evolution and exhibit immune-related features in metastatic colorectal tumors
Source: Cell Death Discov. 2021 Aug 27;7:222. doi: 10.1038/s41420-021-00607-9 (PMC8397721; doi:10.1038/s41420-021-00607-9)
Supplement: Supplementary file 5 — Supplementary figure legends [file 41420_2021_607_MOESM5_ESM.docx]

Figure Legends

**Supplementary figure 1. The arm level CNV events and signaling pathways enrichment in CLM.** A, Exhibition for arm level CNV between primary and metastatic lesions. B, Reactome enrichment of the driver genes in Wnt-β-catanin pathway in CLM (left panel), Model (middle panel) and oncoprints (right panel, only showed the samples with altered) of mutated genes within Wnt_β-catenin pathway model of colorectal cancer. The genes in green is driver, the percentage indicates the mutational frequency. C, Reactome enrichment of liver metastatic genes in HGF/MET pathway (left panel), Model of the frequency of mutation genes in HGF/MET and downstream KRAS-ERK and PI3K-AKT pathway (middle panel) and oncoprints (right panel, only showed the samples with altered in CLM) of recurrently mutated genes within HGF/MET pathway model of colorectal cancer. The genes in green are drivers, the percentage indicate the mutational frequency.

**Supplementary figure 2. Pathway enrichment of mutational genes of S-C clonal pattern.** The genes of clonal models of subclonal-clonal (S-C) from primary to metastasis were enriched significantly in metastasis-related ECM, collagen-containing extracellular, matrix pathways.

Supplementary figure 3. A, Subclonal or clonal TMB loads in primary tumor and liver metastases. B, The difference of total TMB or HLA-corrected TMB between primary tumor and liver metastases.
